# An Energy-Saving Development Initiative Increases Birth Rate and Childhood Malnutrition in Rural Ethiopia

**DOI:** 10.1371/journal.pmed.0030087

**Published:** 2006-02-14

**Authors:** Mhairi A Gibson, Ruth Mace

**Affiliations:** **1**Department of Archaeology and Anthropology, University of Bristol, Bristol, United Kingdom; **2**Department of Anthropology, University College London, London, United Kingdom; BRACBangladesh

## Abstract

**Background:**

Evolutionary life history theory predicts that, in the absence of contraception, any enhancement of maternal condition can increase human fertility. Energetic trade-offs are likely to be resolved in favour of maximizing reproductive success rather than health or longevity. Here we find support for the hypothesis that development initiatives designed to improve maternal and child welfare may also incur costs associated with increased family sizes if they do not include a family planning component.

**Methods and Findings:**

Demographic and anthropometric data were collected in a rural Ethiopian community benefiting from a recent labour-saving development technology that reduces women's energetic expenditure ( *n* = 1,976 households). Using logistic hazards models and general linear modelling techniques, we found that whilst infant mortality has declined, the birth rate has increased, causing greater scarcity of resources within households.

**Conclusions:**

This study is, to our knowledge, the first to demonstrate a link between a technological development intervention and an increase in both birth rate and childhood malnutrition. Women's nutritional status was not improved by the energy-saving technology, because energy was diverted into higher birth rates. We argue that the contribution of biological processes to increased birth rates in areas of the developing world without access to modern contraception has been overlooked. This highlights the continued need for development programmes to be multisectoral, including access to and promotion of contraception.

## Abstract 2

A development programme, in which piped water was supplied to villages, was found to be linked with an increase in both birth rate and childhood malnutrition.

## Introduction

Despite improving living conditions and reduced infant mortality, rural Africa has not experienced the rapid demographic transition to low birth rates that characterize populations in the developed world [ 1, 2]. With the aid of new development initiatives, living and health conditions have been improving and death rates are falling, but birth rates remain high and the population is growing rapidly [ 3]. Africa's population density is projected to double over the next 50 years, rising from 26 to 60 people/km ^2^; over the same interval Europe's will drop from 32 to 27 people/km ^2^ [ 3]. In Ethiopia, the population has been growing by two million a year [ 4]; however, a poor economic growth rate of around 3% per annum [ 5] will sustain existing services for less than a third of the future population. Spiralling population growth and slow economic growth are the main factors fuelling the country's repeated humanitarian crises [ 6].

Global economic events, local culture, and biology can all strongly influence the pace of demographic change [ 7– 9]. The majority of the demographic literature concerns economic models of fertility decline; economic development is seen as the catalyst for declining birth rates [ 8, 10]. However, in the twenty-first century, many of the poorest nations, such as Ethiopia, face slow or stagnant economic growth rates [ 5]. Furthermore, family planning uptake remains low, due to patchy distribution [ 11], poor understanding, and social opposition [ 12– 14]. In rural Ethiopia less than four percent of women use modern forms of family planning [ 4]. In the vast majority of so-called “natural fertility” populations, with limited resources and poor access to contraception, biology may also play an important role in explaining rising fertility [ 15, 16].

Implicit in the debate about international development is that population growth in the developing world is fuelling the demand for family-planning services [ 17]. However, few field studies have adequately addressed the demographic consequences of development intervention in the absence of adequate family planning provisions. Here we investigate how a recent labour-saving technological intervention, which has reduced women's workloads, is contributing to local demographic change in a rural Ethiopian community.

Life history theory is a branch of evolutionary ecology that has been used by evolutionary demographers to explain variation in human fertility and mortality [ 18]. The central tenet of life history theory is that trade-offs exist between available energy allocated to growth, maintenance, and reproduction, and that these trade-offs will be resolved in a way that maximizes fitness [ 19– 21]. Any increased efficiency prompted by a labour-saving intervention technology introduces savings in time and energy that women allocate to work, which can then be reinvested in child care, production, work, and leisure. Life history theory predicts that any increased energetic efficiency for women should increase reproductive function in a resource-limited natural fertility population. Variations in human fertility in response to energetic stress can be seen to represent an evolved feature of women's reproductive systems, designed to enhance lifetime reproductive success by avoiding wasteful allocation of energy to reproductive opportunities with diminished chances of success [ 22, 23].

The field of human reproductive ecology has identified the importance of environmental constraints on reproductive potential [ 15, 22]. A woman's energetic status affects not only the tempo of growth and maturation [ 24], but also measures of fecundity, such as hormone levels during cycling [ 25, 26] and probability of conception [ 27– 29], which mediate the timing of births. Maternal nutrition, workloads, disease, and breast feeding practices play an important role in explaining levels of fecundity. Fertility (number of births) and fecundity (reproductive potential) are obviously related, but the relationship is complex and is mediated by several intervening variables, such as mortality [ 30, 31], morbidity [ 32], and contraception [ 33]. By using demographic, behavioural, and anthropometric data it is possible to explain the causes and consequences of demographic change.

Reductions in women's energetic expenditure have been shown to correlate with higher levels of reproductive hormones [ 27, 34], shorter periods of postpartum amenorrhea [ 35], and lower intrauterine mortality [ 36]. Seasonal peaks in conception rates, associated with changing work patterns, have been identified in Nepal [ 29]; however, no study to date has identified an increase in birth rate resulting from a significant permanent change in women's workloads.

Using life history theory we predict that, in the absence of contraception, a labour-saving development initiative designed to improve maternal and child health may increase fertility. We propose that incremental improvements in health and survivorship associated with development interventions place extra pressures on both individual households and on the overall community through the costs associated with larger family sizes. In a situation of resource scarcity, both mothers and children may experience negative health consequences associated with a pattern of shorter birth spacing [ 36, 37] and increased competition within the household for limited resources [ 38, 39], if contraceptive services are not also provided.

### Study Site

This study is based on an agropastoralist community of Arsi, Southern Ethiopia, which suffers from acute, regular water shortages and chronic food insecurity. Although Arsi is not traditionally considered one of the most drought-prone areas of the country, a declining ratio of land to people, lower land productivity, and low investment in roads and other infrastructure are features prevailing in this region. A survey of relief food requirements in Arsi zones in 1999 estimated that the number of people requiring relief assistance rose dramatically from 35,000 in 1998 to 150,000 in 1999.

In the villages of Hitosa and Dodota subdistricts, there is no free land available for new cultivation, and herding and economic opportunities are limited. This has led to the subdivision of increasingly smaller landholdings, and many new households being offered resettlement outside the region. Access to even the most basic health services, schools, and markets is very limited [ 40]. The nearest health care services and high schools are over 20 km from the villages; however, a planned programme of infrastructural development, including road construction and new government clinics, is expected to improve access to services in the future [ 40]. Contraceptive prevalence is low among rural women (In 2003, fewer than 3% of women had ever used contraception), partly due to a lack of understanding and partly due to a poor supply [ 40]. However, in qualitative interviews, informants suggested that demand for family planning is increasing, which is likely to contribute to an already great need for improved maternal-child health services.

Water shortages, particularly during the dry season months (December to April) can be severe. Mean precipitation is less than 700 mm and there are no perennial rivers. Traditionally, women have borne the brunt of water collection, some transporting the water on their backs in clay pots *(insera)* for distances of up to 30 km. However, between 1996 and 2000 some villages benefited from a water development scheme (the Hitosa Gravity Water Supply Scheme), which has reduced both the energy and time women spent carrying water following the installation of village-level tap stands ( Table 1). Women state their time spent carrying water has been reduced from around three hours to 15 minutes during the driest months. This time is now employed in more social activities, as well as standing in line at the tap stands.

**Table 1:**
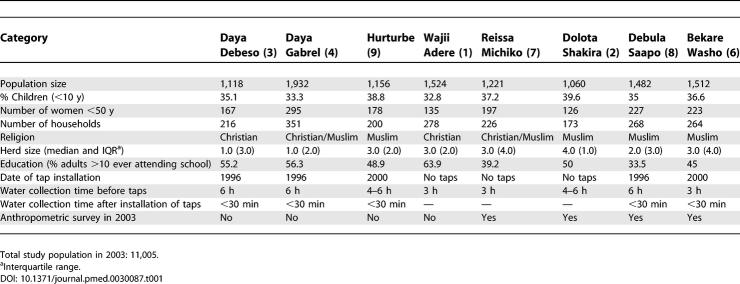
Village Characteristics

In this study, we have compared fertility and child mortality rates both before and after tap installation, and across village, with and without taps, to assess the demographic impact of this technological intervention.

## Methods

Retrospective demographic data were collected between 1999 and 2003 in eight villages, which included those with and without access to the water development scheme. Selection criteria for these villages included comparability of size, altitude, ethnicity, and religion, facilitating comparative analyses both between and within villages ( Table 1). The comparability of villages provided an opportunity for a natural experimental framework. Additional anthropometric and behavioural data were collected across four villages in 2003 ( Table 1).

### Data Collection

A broad demographic survey was completed in all households ( *n* = 1,976), providing data on major socioeconomic and behavioural factors that may influence the demographic process (e.g. household herd size, religion, woman's breast-feeding status). Full retrospective birth histories were collated from 1,548 women of reproductive age (15–49 y) within these households. This included a detailed birth history calendar that recorded the monthly timing of births and deaths over the six years preceding the interview; hence, the periods before and after the water tap installation were included. Each life history event was dated to the year and to the month using a calendar marked with local significant events.

During 2003, a subsample of 682 children (<15 y) and 264 women (15–49 y) were included in an anthropometric survey across four of the villages ( Table 1). This included two villages with, and two without, access to the new water supply. Measures of height and weight were obtained according to the guidelines set out by Lohman et al. [ 41].

### Analyses

The longitudinal demographic data were analysed using discrete-time event history analysis to assess the effects of the improved water supply on birth-spacing and child survival. Event history analysis is a powerful statistical tool for isolating precise demographic effects, e.g., the timing of water development on risk of childbirth and child death. Unlike other standard forms of regression analysis, it can deal with both censored data and time-series data [ 42, 43]. Logistic regression was used to estimate the multivariate models. SAS (Cary, North Carolina, United States) software version 8.2 was used to perform the statistical analyses.

Independent variables controlled for, in the models of relative probabilities of both childbirth ( Figure 1) and child death ( Figure 2) over time, included mother's age, parity, education, and socioeconomic status. Since the exact timing of the tap installation was known, water point access was entered into the model as a time-varying covariate. Mortality and fertility interrelatedness [ 38, 44] were controlled for in each model. An early child death and cessation of the amenorrhoeic effects of breastfeeding increased the risk of birth per month; conversely, shorter birth-spacing, which may have introduced sibling competition, increased a child's risk of dying under three years of age.

**Figure 1:**
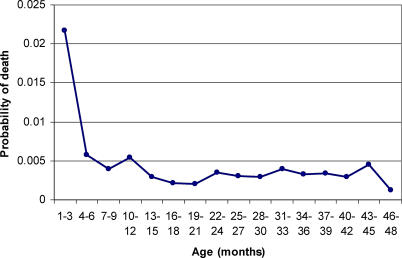
Probability of Child Death by Age (0–4 y of Age; 2,914 Births)

**Figure 2:**
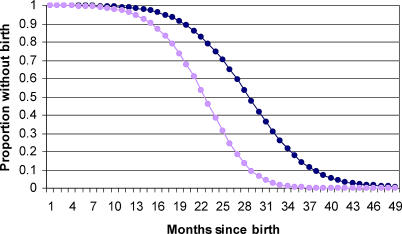
Model of Risk of Birth over Time by Water Access Data presented are controlled for child survivorship, mother's age, parity, religion, socioeconomic status, and village for 1,526 women. Purple curve, access to taps; blue curve, no access to taps.

Controls for time (in months) and time-squared were entered in the models, since the risk of both birth and death vary as a nonlinear function of length of exposure. Risk of birth has a curvilineal, “∩”-shaped relationship with time and is greatest at 18–36 mo postpartum [ 15]. Among the Oromo sample, risk of dying was greatest during the first few months of life ( Figure 1). Separate models (unpublished data) were run to explore interaction effects between length of exposure (time since birth) and each of the independent variables. The only significant interaction term identified, between time and water point access, was included in the final models.

The dependent variable, monthly probability of birth, was modelled up to four years postpartum ( Table 2). The dependent variable, monthly probability of death, was modelled up to the third year of life, since the greatest risk of child death for this population was considered to occur during the first few years of life ( Table 3). To control for any hierarchical structures in the data relating to the household (genetic and/or environmental effects within family), only the last two birth intervals per women were included in the fertility analyses, and only last two births per woman were included within the mortality analyses. A total of 1,548 noncontracepting women contributed 2,914 births for the final analyses.

**Table 2:**
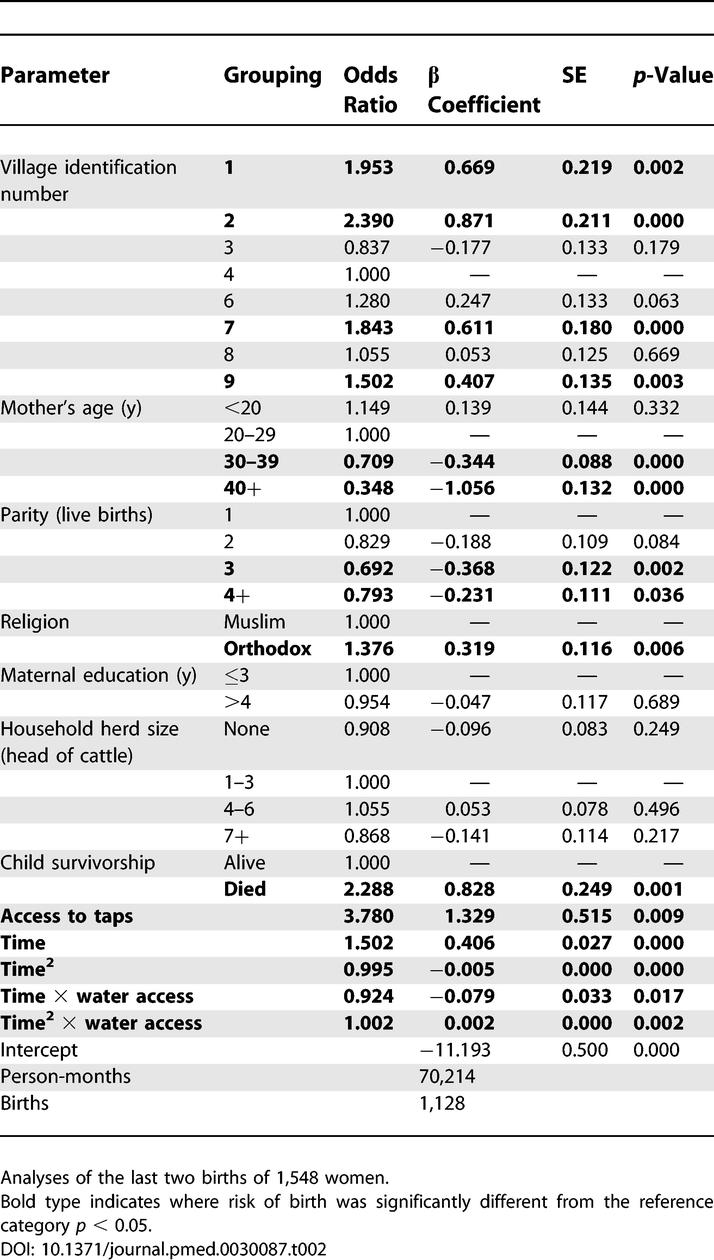
Multivariate Event History Regression Model for Monthly Risk of Birth

**Table 3:**
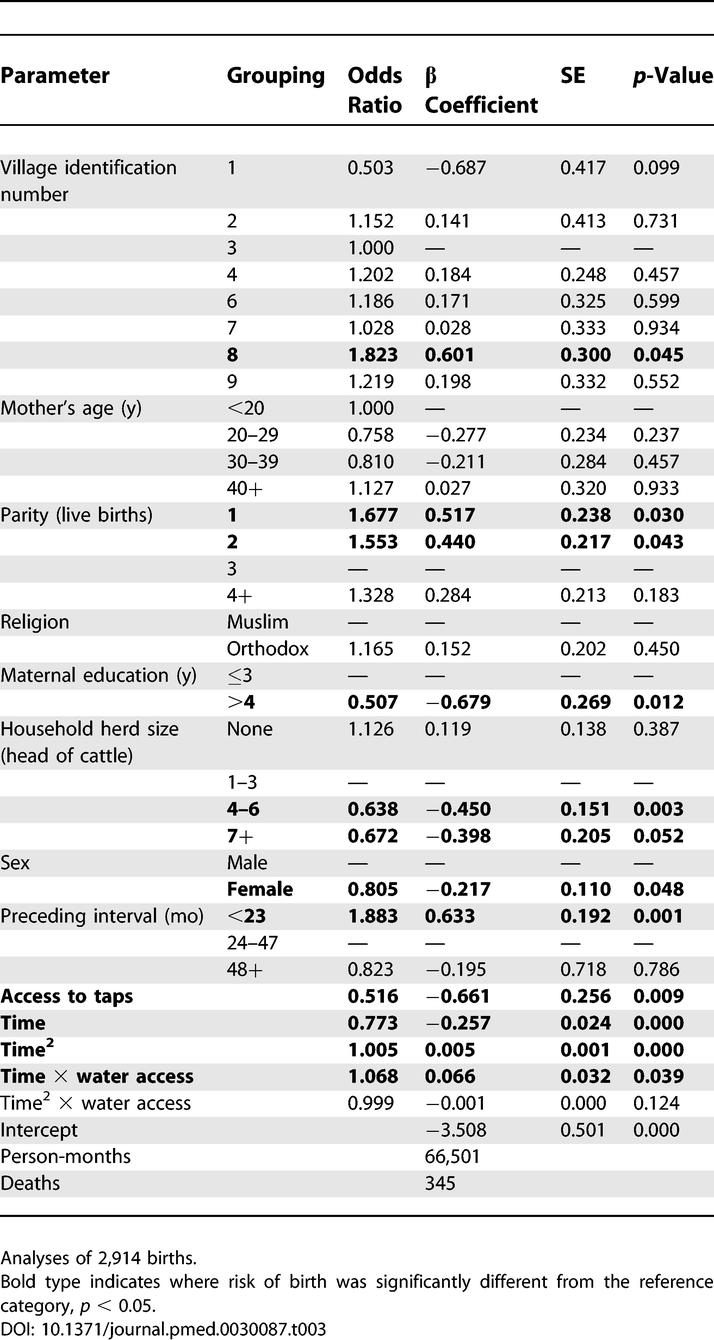
Multivariate Event History Regression Model for Risk of Dying before 3 y of Age

In the cross-sectional anthropometric analyses, multivariate general linear models (GLMs) were employed to assess the partial effects on child nutritional status of child's age, maternal age, sex of the child, and birth order, as well as a covariate for presence of village water points at the survey date; these results are presented as Z-scores for weight for age (WAZ), height for age (HAZ), and weight for height (WHZ). Standardized Z-scores, or standard deviations, represent the width of the distribution around the mean of the reference population for children of the same age and sex. By convention, children with a WAZ of more than two Z-scores below the median of the World Health Organization/National Center for Health Statistics international reference population are considered seriously or acutely malnourished [ 45]. Children with a HAZ more than two Z-scores below the reference median are considered seriously or acutely stunted. The standardized scores control for age- and sex-specific patterns of growth and allow direct comparisons of children of all ages and both sexes [ 45, 46]. Multivariate models were also built to identify predictors of maternal nutritional status (both body mass index [BMI] and mid-upper arm circumference [MUAC]) for the sample adult population. GLMs provide adjusted means and standard errors. SPSS (Chicago, Illinois, United States) software version 12.0 was used to perform the statistical analyses. Z-score values based on the World Health Organization/Centers for Disease Control international reference population [ 45, 46], were calculated using Epi-Info 3.2.2 software (Centers for Disease Control and Prevention; available at http://www.cdc.gov/epiinfo).

## Results

### Fertility Analyses

Improved water access was associated with increased monthly relative risk of childbirth ( Figure 2). The odds of a woman with access to water points experiencing a birth in any given month was three times greater than a woman without an improved water supply ( Table 2; access to taps odds ratio [OR] = 3.78, *p* = 0.009). A significant interaction term between water access and time, indicated that the effects of the water-point function on risk of birth varied over the months since birth (time × water access OR = 0.924, *p* = 0.017; time ^2^ × water access OR = 1.002, *p* = 0.002). Relative monthly risk of birth assumed a curvilineal, “∩”-shaped relationship with time, the water points having the greatest influence on fertility during the women's most fertile months, 18–36 mo postpartum.

There were a number of other significant predictors of women's birth interval length, reflected in their monthly risk of birth over time ( Table 2). The death of the previous child was a very strong positive predictor of a women's risk of birth; early child death was associated with a premature cessation of breastfeeding, resumption of menses, and early subsequent birth. Maternal age, parity, and religion also strongly influenced a mother's monthly risk of birth. Older women and those of high parity had lower relative risk of birth than younger women with lower parity. Religion also significantly predicted birth-spacing. Christian Ethiopian Orthodox women had a higher risk of birth than Muslim women; possibly relating to a cultural preference for a shorter period of postpartum sexual abstinence.

### Mortality Analyses

Improved access to water was associated with a reduced relative risk of child death per month ( Figure 3). Monthly risk of child death was 50% lower with access to new tap stands ( Table 3; OR = 0.516, *p* = 0.009). The accelerated risk of dying under conditions of poor water availability was most apparent during the first year of life; however, the absolute benefit of water availability was retained up to 39 months ( Figure 3).

**Figure 3:**
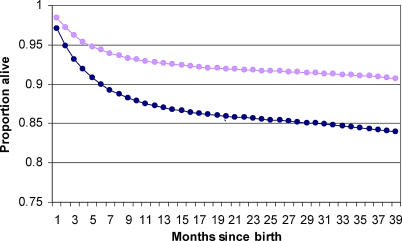
Model of Risk of Death over Time by Water Access Data presented are controlled for sex, preceding interval length, mother's age, parity, religion, socioeconomic status, and village for 2,914 births. Purple curve, access to taps; blue curve, no access to taps.

The analyses identified other significant predictors of child survival ( Table 3). Birth-spacing patterns were a good predictor of child survival. Shorter birth interval length (<23 mo) was associated with increased risk of child death for the subsequent child. Low birth-order children were at a higher relative risk per month of dying than children at higher parity. High socioeconomic status households had relatively lower risk of experiencing an early child death: children whose mothers had more than four years of formal schooling and those in households with the largest herd sizes (more than four cattle), had higher odds of surviving early childhood. Female children experienced a lower risk of death than male children.

### Health and Nutrition Analyses

The analyses provided no evidence to indicate that development intervention had improved maternal and child health. In 2000, one-fifth of all women (21%) were chronically malnourished (BMI < 18.5; *n* = 464); however in an anthropometric survey undertaken three years later 17% of women still had a BMI below 18.5 [ 40]. In 2003, access to water taps did not predict maternal body fat levels. Estimated marginal means across the villages, controlling for maternal age, parity, lactation status, and household herd size, indicated that there were negligible differences in BMIs or MUACs across villages with differing access to taps ( Table 4).

**Table 4:**
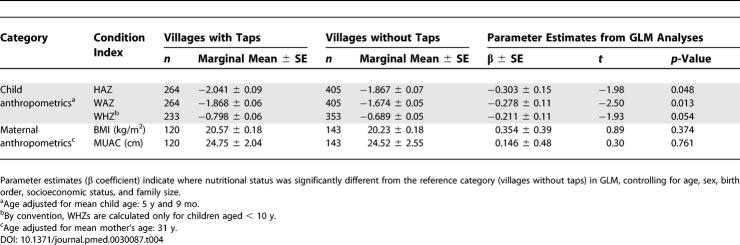
Child and Mother Anthropometrics by Village Water Source

A third of all children under 15 years ( *n* = 684) were seriously malnourished [WAZ < −2.00] and a half were seriously stunted [HAZ < −2.00]. Around 2% of children were severely wasted [WHZ < −3.00], the cut-off used to define children at a high risk of morbidity and mortality in therapeutic feeding programmes [ 45]. Access to a water tap at the survey date was negatively associated with child nutritional state ( Table 4). After controlling for any effects of age, sex, birth order, socio-economic status and family size, children living in villages with water access were at a significantly greater risk of being malnourished (low WAZ; GLM β coefficient = −0.278 ± 0.11, *t* = −2.50, *p* = 0.013) and stunted (low HAZ; GLM β coefficient = −0.303 ± 0.15, *t* = −1.977, *p* = 0.048). However, there were interesting age effects across the villages. Figure 4 illustrates the median WAZ for all children below ten years of age in villages with and without taps. This plot reveals that most of the variation in nutritional levels associated with differing access to taps was among children below the age of 5 y; children at older ages had similar weight (and height) across the villages. Since most taps were installed 3–6 y before the anthropometric survey ( Table 1), this indicates that only children who had been born since water tap installation had lower levels of nutritional status.

**Figure 4:**
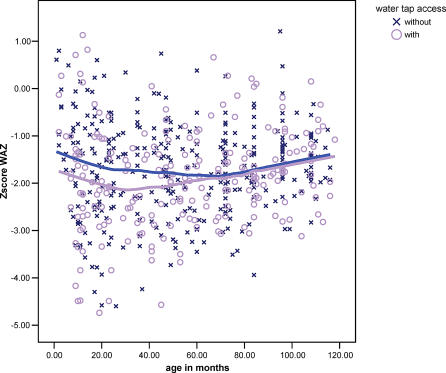
Z-Scores for Weight for Age in Children Aged 0–10 y by Water Tap Access Dataset includes *n* = 350 without taps and *n* = 227 with taps.

There is no evidence to suggest that women had altered their breast feeding practices following tap installation, since no differences in median duration of breast feeding were observed between women in villages with and without taps in 2003 ( Table 5). A health survey undertaken in the villages, in which women reported any ill health they had experienced over the two weeks preceding the interview and their health-seeking behaviours, indicated that there were no significant differences in reported ill health or contraceptive uptake across the villages, although women in villages with new taps did appear to visit a health clinic when ill ( Table 5) [ 40].

**Table 5:**
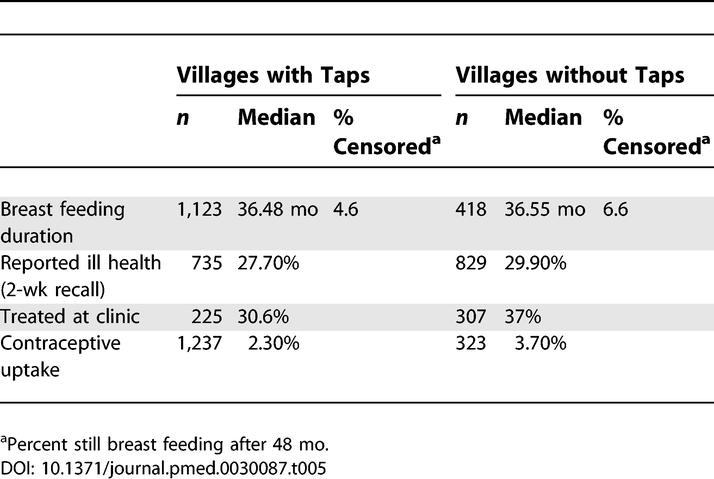
Breastfeeding Practices and Health of Women by Village Water Source

## Discussion

This study is the first, to our knowledge, to demonstrate a link between a specific technological intervention and an increase in birth rate and decrease in mortality at the village level. The increased birth rate is likely to be mediated by improvements in women's workloads, brought about by reducing energetic expenditure on water collection, since women's nutritional levels, breast feeding practices, and health do not vary ( Table 5). Female health outcomes (nutritional status and morbidity levels) do not appear to be influenced by reduced workloads, lending support to the idea that women's surplus energy is diverted towards reproduction. Increased levels of child survival are likely to relate to improvements in the quality and quantity of water supply [ 47, 48] and greater opportunities for direct maternal childcare. We propose that the energy saved by a new development technology is being diverted to enhance fertility and reduce mortality; however, since the underlying resources in the system are limited, this comes at the cost of an increase in childhood malnutrition.

Higher birth rates not counterbalanced by mortality are likely to be fuelling population growth in this rural population. Furthermore, larger family sizes may increase childhood malnutrition in a population already at carrying capacity [ 49, 50]. There are two possible explanations for the increase in levels of malnutrition, which may relate to improved levels of child survival in villages with access to taps: higher competition between siblings for limited resources, and/or relaxed selection against low birth-weight babies. In this case, the latter seems the most likely explanation: If siblings were competing with one another for household resources, then poor nutritional status should be recorded across children of all age groups; instead, higher levels of malnutrition are found only among children born into conditions of improved water supply (those below the age of 5). We propose that, due to enhancements in maternal energy budgets associated with the labour-saving development intervention, smaller, low birth-weight offspring are coming to full term and surviving critical periods of early childhood. Other studies examining the impact of health interventions in natural fertility populations have found no improvement in child growth patterns, possibly for similar reasons; for example, attempts to reduce diarrhoea prevalence did not improve child nutritional status in either rural Gambia or Bangladesh [ 51, 52].

Whilst recent demographic literature has focused on issues relating to low fertility [ 53] or HIV/AIDS, the problems of rapid population growth have not disappeared for much of rural Africa [ 1]. Evidence based on country-level statistics suggests that during demographic transitions, countries undergoing mortality declines do experience an initial rise in birth rates prior to a long-term decline in fertility [ 54]. However, in most of these cases the reduction in mortality has arisen from endogenous, gradual economic developments [ 2]. Instances where mortality decline is brought about by external intervention in the absence of general economic improvement may not necessarily follow the same pattern. In Ethiopia, in the current climate of declining mortality and slow national economic growth [ 5], higher birth rates are not sustainable. A recent UN report highlighted the country's spiralling rural population growth, slow economic growth, and environmental degradation as the main factors fuelling Ethiopia's near perpetual humanitarian crises [ 6].

Further increases in the Oromo villages population may increase the competition for limited resources, placing increased pressure on existing community services and ultimately, fuelling the trend towards rural-urban migration. In recent years, the lack of land available for new cultivation or herding and poor economic opportunities have led to the subdivision of landholdings, and many new households are being offered resettlement outside the region. Many landless young men and women are likely to seek employment in the towns and cities; currently, up to 50% of the urban population are migrants from such rural villages [ 55].

Improvements brought about by the water development scheme have included increased leisure time for women and reduced childhood mortality. However, the findings of this study also highlight that development intended to improve human welfare that does not include a family planning component can actually undermine the long-term well-being of the target population [ 49, 50]. Incremental reductions in women's workloads caused by technological advances may be exerting unexpected pressure on families in the developing world through the costs of higher fertility. Whilst it is possible that these stresses, over the longer term, will drive a desire for smaller family size, many rural populations such as this one remain without easy access to family planning services. This study highlights the need for development programmes to be multisectoral, including components that promote and improve access to contraception.

**Patient Summary**

**Background**

Development programmes in low-income countries, for example in Africa, often focus on the welfare of women and children. This approach is desirable for humanitarian reasons, but there are also held to be other benefits (for example to the economy), and it is widely believed that if child death rates can be reduced people will choose to have fewer children. In rural Africa, however, even where child death rates have declined, birth rates have not necessarily fallen. On the basis of current trends, it is predicted that Africa's population will double in the next 50 years. The resources available to Africa would not be able to sustain so many people. The situation in Ethiopia provides an illustration; spiralling population growth and slow economic growth are the main factors fuelling this country's repeated humanitarian crises.

**Why Was This Study Done?**

Can some development programmes in Africa fail to make sustainable improvements over the long run, because they lead to unsustainable increases in population? The researchers studied a programme intended to improve the lives of Ethiopian women; they measured its impact on the health of the women and their children, and also on the birth rate.

**What Did the Researchers Do and Find?**

The programme they studied involved a rural area where some villages had benefited from a tapped water supply. Previously, women had to walk long distances (up to 30 km) to fetch their families' water in clay pots. The development project reduced the time they spent carrying water each day from around 3 hours to about 15 minutes. The researchers had access to information over a 4-year period, including both villages where tapped water had been introduced and others where it had not. In total nearly 2,000 households were included. The nutritional status of the women and children (in terms of each person's weight and height) was also known. The availability of tapped water improved the survival of young children, although the nutritional status of the women and the nutritional status of young children actually declined. And the birth rate increased. All this caused greater scarcity of resources within households.

**What Do These Findings Mean?**

The researchers believe that the energy the women saved by not having to carry water was “diverted into higher birth rates.” They argue that development projects that focus on just one issue (in this case water supply) can cause long-term problems and that it is preferable for development to be “multisectoral.” Improving access to contraception, which was poor in the area where this study was done, should be a key part of development programmes.

**Where Can I Get More Information Online?**

Numerous government agencies and nongovernment organisations are active in development programmes in low-income countries. Many of them focus on health and some on population issues. One example is Interact Worldwide:

http://www.populationconcern.org.uk

An overview of the issues concerning population and development may be seen on the Web site of the United Nations Populations Fund:

http://www.unfpa.org

The United Nations Population Fund publishes an annual report, State of World Population: http://www.unfpa.org/swp/2005/english/ch1/index.htm

## Abbreviations

BMI: body mass index
GLM: general lineal model
HAZ: height for age Z-score
MUAC: mid-upper arm circumference
OR: odds ratio
WAZ: weight for age Z-score
WHZ: weight for height Z-score
